# Research activity and capacity in primary healthcare: The REACH study: A survey

**DOI:** 10.1186/1471-2296-10-33

**Published:** 2009-05-11

**Authors:** Liam G Glynn, Ciara O'Riordan, Anne MacFarlane, John Newell, Alberto A Iglesias, David Whitford, Peter Cantillon, Andrew W Murphy

**Affiliations:** 1 Department of General Practice, National University of Ireland (NUI), Galway, Ireland; 2 Clinical Research Facility, NUI, Galway, Ireland; 3 Department of Mathematics, NUI, Galway, Ireland; 4 Department of Family Medicine, Royal College of Surgeons in Ireland – Medical University of Bahrain, PO Box 15503, Adliya, Kingdom of Bahrain

## Abstract

**Background:**

Despite increased investment in primary care research and development (R&D), the level of engagement of primary healthcare professionals with research remains poor. The aim of this study is to assess the level of research activity and capacity for research among primary healthcare professionals in a health authority of over one million people in a mixed urban/rural setting in the West of Ireland.

**Methods:**

A questionnaire, incorporating the R+D Culture Index, was sent to primary healthcare professionals in the HSE Western Region. Baseline characteristics were analysed with the use of one-way ANOVA and Chi-square test and the dependence of R&D Culture Index score on all sixteen available covariates was examined using multiple regression and regression tree modelling.

**Results:**

There was a 54% response rate to the questionnaire. Primary healthcare professionals appeared to have an interest in and awareness of the importance of research in primary care but just 15% were found to be research active in this study. A more positive attitude towards an R&D culture was associated with having had previous research training, being currently involved in research and with not being a general practitioner (GP) (p < 0.001), but much variability in the R&D culture index score remained unexplained.

**Conclusion:**

Despite awareness of the importance of R&D in primary care and investment therein, primary healthcare professionals remain largely unengaged with the R&D process. This study highlights the issues that need to be addressed in order to encourage a shift towards a culture of R&D in primary care: lack of research training particularly in basic research skills and increased opportunities for research involvement. The use of the R&D Culture Index may enable groups to be identified that may be more research interested and can therefore be targeted in any future R&D strategy.

## Background

There is a growing body of evidence demonstrating that the cost-effectiveness of any national health care system is strongly correlated with the strength and position of primary care within that system[1,2]. To enhance primary care practice, there has been an increasing emphasis on clinical decision making which is based on sound research evidence. That evidence must come at least in part from research carried out within the primary care setting [2]. In addition, when those working in primary care are involved in research and development (R&D), it has been shown that there is an increase in the quality of care provided as well as faster dissemination and adoption of research evidence[3].

However, the level of engagement of primary care practitioners with research has been traditionally poor [4-6]. The reasons for this are unclear, but attempts to promote a culture of R&D continue to be hindered by the prevailing perception of research as a remote science and the absence of a supporting infrastructure[5,7,8]. Historically research in primary care has been under-resourced and marginalized over a prolonged period of time[2,9]. This has changed more recently with increased funding and support for primary care research in countries such as the UK but the level of engagement of primary healthcare professionals in research activity appears to have remained static during this period[6,7,9,10]. The Republic of Ireland, where this study took place, lags behind the UK in terms of investment in, and outputs from, primary care R&D[2].

The aim of this study was to assess the current level of research activity and research culture of primary healthcare professionals in a large health authority and to examine the factors affecting aptitude for future research through use of the R&D Culture Index. The R&D Culture Index has been developed to identify personal and organisational development needs and to guide strategy in advancing practitioner engagement with research[6]. However, use of the R&D Culture Index to date has been confined to single professional groupings[11,12] or has been limited by poor statistical methods[6,11]. 'Research culture' is used in this context to refer to the capacity of practitioners to engage in R&D activity.

## Methods

Primary healthcare professionals of the following professional groups working within the Health Services Executive (HSE) Western Region (a health authority of over one million people in a mixed urban/rural setting in the west of Ireland) on June 1^st^, 2006 were included in the study: community pharmacists; general practitioners (GPs); practice nurses; primary health care managers; public health doctors; public health nurses. Other primary health care professionals in the community were not included in the study due to absence of a centralized database for contact details. Contact details for all of the above primary health care professionals, with the exception of practice nurses, were obtained from local primary care units. Practice nurses are not contracted to the HSE but rather are employed directly by practices and so were contacted through their respective practices. All primary health care professionals were sent a piloted postal questionnaire with a covering letter and stamped addressed envelope. All non-responders were sent a second questionnaire after 4 weeks. This mailing strategy followed the Dillmann techniques[13] in an attempt to maximise response rates among participants.

The postal questionnaire used in the study consisted of three sections. Section A gathered demographic details on participants such as age, gender, qualifications, level of clinical experience, research training and current work status. Section B consisted of the "Research & Development (R&D) Culture Index", a recently developed and validated research tool which is used to assess capacity for and attitude towards research and development among healthcare professionals[6]. Section C assessed level of research engagement and research training needs among participants as well as perceived barriers and levers to participation in research. The R&D Culture Index is an 18-item questionnaire, scored on a four point likert scale from strongly disagree to strongly agree (0–3). Possible scores on the R&D Culture Index range from 0–54 with higher scores indicating a more positive attitude towards an R&D culture. In addition, respondents are asked to identify the five items that they perceive to most strongly contribute to an R&D culture. The statements address three specific domains: personal skills and aptitude towards R&D; working environment facilitatory towards R&D; and organisational infrastructure encouraging R&D. Previous validation has demonstrated a Cronbach alpha coefficient of 0.92 indicating good internal consistency for the whole index[6].

Baseline characteristics of study participants were analysed with the use of one-way ANOVA for continuous variables and Chi-square test for categorical variables. The primary aim in the analysis was to model the dependence of R&D Culture Index Score on the following sixteen explanatory variables: age, gender, work status, professional group, professional registration, number of years registered, postgraduate qualification, higher training qualification, research training, time in post, research involvement, named applicant on research funding application, named applicant on research ethics application, author of peer-reviewed publication, author of non-peer reviewed publication, presenter of research conference paper. Two different statistical approaches were used; multiple regression (using variable selection techniques) and regression tree modelling. The choice of best model amongst the candidate models was determined using best subsets and stepwise regression[14]. In the tree based analysis the final regression tree derived employed the CART approach[14]. This involved successive binary partitioning of the data set by identifying at each partition the explanatory variable which maximized the between-groups sum-of-squares using analysis of variance. Pruning was based on examining the complexity parameter and cross validation costs using the 1-SE rule[15]. All statistical test values were two-sided, and a P value of less than 0.05 was considered to indicate statistical significance. Analysis was carried out using SPSS (14.0) and *R *statistical software. Ethical approval was granted by the research ethics committee of the Irish College of General Practitioners.

## Results

### Subjects

Excluding practice nurses, there were 733 relevant primary health care professionals working within the HSE Western Region on June 1^st^, 2006. There were a total of 498 respondents to the survey. This figure included 100 responses from practice nurses, for whom it was not possible to calculate a denominator due to the absence of a local or national register of practice nurses. Excluding practice nurses, the overall response rate was 54% (398/733). The mean age of respondents was 45.6 years (SD = 9.1) with over half (57.4%) being female and the majority (81.7%) working full time. One third (33.5%) of respondents had been in their current post for five years or less. Only 12% had a postgraduate qualification but 42% had received formal research training of some kind. Respondents were grouped according to profession into general practitioners [GPs] (n = 286); nursing staff [public health nurses and practice nurses] (n = 170); and other HSE staff [health managers, community pharmacists and public health doctors] (n = 42). Table 1 illustrates the baseline characteristics of the study participants, and R&D Index Score, according to professional group. GPs were comparatively older, predominantly male, working full-time, longer in post and less likely to have received formal research training. Nursing staff were predominantly female and more than half had formal research training. Nurses were least likely of the professional groups to have a postgraduate qualification while over 40% worked only part-time. HSE staff were also predominantly female while nearly half had a postgraduate qualification and the majority had formal research training.

**Table 1:** Baseline characteristics of 498 primary healthcare professionals according to professional group

**Characteristic**	**General Practitioner** **(n = 286)**	**Nursing** **(n = 170)**	**HSE Staff** **(n = 42)**	**P-value**
**Mean Age in years (SD)**	47.8 (8.8)	42.7 (8.9)	42.6 (7.8)	**< 0.001**
**Female sex (%)**	29.2	99.4	80.5	**< 0.001**
**Working full time (%)**	94.4	59.5	85.4	**< 0.001**
**Years in Current Post (SD)**	16.0 (9.7)	8.2 (7.5)	7.2 (8.4)	**< 0.001**
**≤ 5 years in current post (%)**	19.4	49.7	65.0	**< 0.001**
**Formal Research Training (%)**	31.0	57.1	59.0	**< 0.001**
**Postgraduate qualification (%)**	11.0	5.0	45.7	**< 0.001**
**R&D Index Score**	29.1 (6.3)	33.4 (6.8)	34.8 (7.2)	**< 0.001**

### Research and Development Culture

The responses to the eighteen statements in the R&D culture index are shown in Table 2 in order of highest level of agreement. Agreement with the statements ranged from 43% to 86% of respondents. Respondents appeared to suggest that the structure and leadership required for a culture of R&D was poor with only 54% agreeing there was strong professional leadership in this area. In addition, less than half of respondents agreed that there were regular staff meetings to explore ideas (43%) or that it was possible for them to organise their own time to create opportunities to develop professional practice (48%). However, there appeared to be both a high level of awareness of the influence of research on professional practice (83%) and a widespread desire to share professional practice development ideas/research/information across the health service (86%). In addition, the greater majority of respondents stated that they were very keen to use research in their professional practice (73%) and stated that they would like to learn more about research activity during the next 6 months (73%). Respondents were then asked to select five out of the eighteen statements in the R&D culture index which they felt would be most crucial in facilitating a R&D culture within their own working environment. The final column of table 2 describes the resultant ranking of the eighteen statements of the R&D culture index in terms of their importance in facilitating a research and development culture. Four out of the five highest ranked statements relate to respondents' working environment and organisational infrastructure as opposed to personal skills and attributes which were ranked relatively lower, occupying four of the five lowest ranked statements.

**Table 2:** Results of the Research & Development Culture Index (in order of highest agreement)

Domain*	**Questionnaire Item**	**Agree (%)** **(n = 437–460)**	**Ranking for facilitation of R&D Culture** **(n = 437) (%)**
**PERSONAL**	I would like more opportunities to share professional practice development ideas/research/information across the Health Board	393 (86%)	**5 **(33)
**PERSONAL**	I know how professional practice is influenced by research	378 (83%)	**17 **(17)
*ORGAN*.	I have the skills to use library and learning facilities	367 (80%)	**7 **(30)
WORK	If I have an idea to improve clinical or work practice, I have the knowledge and skills to address it	350 (77%)	**9 **(28)
WORK	There are opportunities to reflect on my work/practice	346 (76%)	**4 **(36)
WORK	Development of my professional practice/work is valued as part of my job	337 (74%)	**3 **(39)
WORK	There is an opportunity to develop professional practice in my area	329 (73%)	**10 **(27)
**PERSONAL**	I am very keen to use research in professional practice	328 (73%)	**12 **(21)
**PERSONAL**	I would like to learn more about research activity during the next 6 months	327 (73%)	**15 **(19)
**PERSONAL**	I understand research terminology	282 (61%)	**14 **(20)
*ORGAN*.	There are people around to help and support me to change and develop professional practice	246 (54%)	**2 **(50)
WORK	My discipline here works as equal partners with other disciplines to change or develop professional practice	245 (56%)	**18 **(17)
*ORGAN*.	There is strong professional leadership	242 (54%)	**13 **(20)
*ORGAN*.	I have access to training and development opportunities which give me the skills to question and investigate practice	238 (53%)	**1 **(52)
WORK	The development work that I do links with the plans of the Health Service Executive and the Primary Care Strategy	227 (52%)	**11 **(22)
**PERSONAL**	I feel confident about using research in my professional practice	219 (48%)	**16 **(19)
**PERSONAL**	I can organise my own time to create opportunities to develop professional practice	216 (48%)	**8 **(29)
WORK	There are regular staff meetings to explore ideas	195 (43%)	**6 **(30)

*The domains of the research and development culture index are:

WORK *= Working environment facilitatory towards R&D*

ORGAN. = Organisational infrastructure encouraging R&D

**PERSONAL **= *Personal skills and aptitude towards R&D*

### Research activity

When participants were questioned about involvement with research, 15% reported being currently involved and the majority reported either current or former involvement. In the previous twelve months, 4.5% (22/492) had been named on a research funding application; 4.3% (21/492) had been named on an research ethics committee application; 3.3% (16/492) had been an author of a peer reviewed research publication; 3.3% (16/492) had been an author of a non-peer reviewed research publication; and 7.5% (37/492) had presented a conference research paper. HSE staff were significantly more likely than GPs to have been named on a research ethics committee application (χ^2 ^= 18.3, 2 df, p < 0.001); been an author of a peer reviewed research publication (χ^2 ^= 12.2, 2 df, p = 0.002); or presented a conference research paper (χ^2 ^= 54.8, 2 df, p < 0.001). Nursing staff were significantly less likely than either HSE staff or GPs to be involved in such activities.

### Barriers to research involvement and research training

When asked what factors prevented or precluded research involvement or interest, participants reported lack of protected time at work (82%), lack of funding (53%), lack of training (48%), lack of knowledge/research skills (43%) and lack of supervision or support (41%). Clinical staff (GPs and nursing staff) identified lack of protected time (p = .001) and lack of funding (p = .004) as barriers to R&D more frequently than other HSE staff. In terms of promoting personal research involvement, participants placed greatest value on protected time (64%), as well as availability of expert support and supervision (56%), support of a peer group (52%) and training in specific research skills (47%). The research skills training need identified by participants most frequently was in basic research skills.

### Research and Development Culture Index Scores

Of the total group, 359 (72%) participants completed the R&D Culture Index giving a mean score of 31.1 (SD = 6.9) with a minimum score of 14 and a maximum score of 53. Mean scores differed significantly (F = 22.08, p < 0.001) across the professional groups with GPs scoring lowest (with a mean score of 29.1). A comparatively higher score on the R&D Culture Index was also associated with being female (t = -3.80, p < 0.001); having a postgraduate qualification (t = -2.83, p = 0.005); being in current post for 5 years or less (t = 3.42, p = 0.001); being part-time (t = -2.29, p = 0.023); having had previous research training (t = 6.83, p < 0.001); and having had some involvement with a research project in the past (t = -2.64, p = 0.009).

**Figure 1:**
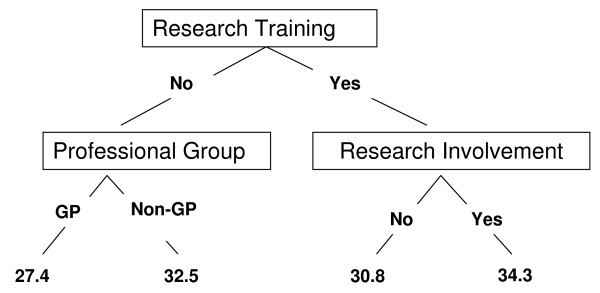
**Regression Tree for predicting R&D Culture Index Score with mean score (and number of respondents) given at the terminal nodes**. The statistical modeling used in the above analysis adjusted for the following covariates: age, gender, work status, professional group, professional registration, postgraduate qualification, higher training qualification, number of years registered, research training, time in post, research involvement, named applicant on research funding application, named applicant on research ethics application, author of peer-reviewed publication, author of non-peer reviewed publication, presenter of research conference paper. (professional group, research training and research involvement were the covariates kept in the final multiple regression model and the final tree regression model).

The best regression model identified three significant explanatory variables which explained the dependence of R&D Culture Index score on the available covariates using both statistical approaches: 'research training', 'research involvement' and 'professional group' (r^2 ^adjusted = 23%). The regression tree approach identified two different trees as potentially optimal (on pruning); one (r^2 ^= 20%) had the identical explanatory variables as identified using multiple regression [see Figure 1], [ie: 'research training', 'research involvement' and 'professional group'] while the other tree (not illustrated in figure 1) (r^2 ^= 20%) included 'presenter of research conference paper' rather than 'research involvement'.

There was convincing evidence of a significant association between both conference attendance and research involvement (p < 0.001 in both cases) suggesting that the trees are essentially returning the same information. The Tree based model is more efficient in dealing with missing values and given that the two approaches identify a nearly identical subset of useful explanatory variables, the possibility that the missing values are having an undue influence on the final regression model is remote. The amount of variability in R&D Culture Index score explained by the regression model is relatively low (r^2 ^adjusted = 23%).

## Discussion

### Summary of main findings

This study shows that the primary healthcare professionals in this large healthcare authority appeared to have an interest in and awareness of the importance of research in primary care. In a country such as Ireland with an underdeveloped culture of primary care R&D, 15% of primary healthcare professionals remain research active at any one time. However, almost a third of respondents had never been involved in a research project and 90% of respondents had not achieved any 'research milestones' in the past 12 months. This appears to be primarily due to lack of research training particularly in basic research skills. However, 'research involvement' also appears as a strong predictor of a higher R&D Index score. Increased opportunities for research involvement may therefore influence research activity and capacity but such opportunities remain dependent on issues such as training, protected time and funding. A striking result was that GPs, the largest professional group in primary care, appear to have a more negative perception of R&D culture than the other professions in this study. Groups with a more positive perception of R&D culture are more likely to carry out research and so may merit identification and specific support and training when trying to encourage research activity. However, a perceived lack of confidence about using research in practice is apparent. This confidence may be improved by undergoing training in research skills and by increased exposure to a culture of R&D through attendance at conferences and peer research groups. Indeed, the factor most commonly regarded as essential for facilitating a R&D culture was the availability of training and development opportunities, but only about half of the respondents felt that these opportunities were actually available.

### Strengths and limitations of this study

This study presents the first available evidence in regard to research activity and capacity among a representative sample of primary healthcare professionals in the Republic of Ireland. Our study had a number of limitations. Firstly, direct contact details for practice nurses were not available as they are not contracted to the HSE but rather are employed directly by practices and so there was no information available for non-responder practice nurses. Secondly, the authors were not able to include in the study other primary health care professionals in the community. Thirdly, the response rate in this study was 54%, thereby limiting external validity. Characteristics of the source population in its entirety could not be established and therefore there is no evidence that the sample in this study is representative of the entire primary healthcare professional population. However, this response rate is comparable to, or better than, other studies using the R&D culture index. Finally, the amount of variability in R&D Culture Index score explained by the statistical models was quite low (r^2 ^adjusted = 23%), suggesting that R&D Culture Index may not be a precise metric for research activity or that other important factors in determining research activity and capacity were not measured.

### Comparison with existing literature

Primary healthcare professionals in a primary care Authority in the UK[6] and nurses in an integrated Authority in Northern Ireland[11] prioritised the same contributors to the development of a culture of R&D as in this study. They highlighted the importance of organisational infrastructure and an appropriate working environment in developing research capacity as opposed to personal skills and attributes which were ranked relatively lower, occupying four of the five lowest ranked statements. The proportion of respondents with formal research training (42%) was similar to that reported in a study carried out nearly ten years previously in the UK[10] (38%), probably reflecting the underdeveloped position of primary care R&D in this country.

The proportion of primary healthcare professionals involved in research at any one time appears remarkably consistent when compared to international literature. The figure in this study (15%) is similar to that reported previously[6,7,10]. However, the ability to publish in peer-reviewed journals over a twelve month period (3%) was lower than that previously reported by Whitford[6] (4%), Robinson[10] (4%) and Jowett[7] (9%). The Mant report[2] confirmed this under-performance in terms of output in Irish primary care compared to the UK despite the fact that, as this study suggests, a similar proportion of primary healthcare professionals appear to be involved in research in both settings. It is likely that the major drivers of research output and the major R&D supports for primary healthcare professionals do not arise from within primary care but from external sources such as government, universities and funding bodies.

### Implications for future research and clinical practice

The continued low level of interaction of GPs with primary care research is worrying as it will contribute further to the decline in enquiry-led research[16]. The Mant report[2] suggested that Ireland was underperforming in the conduct of R&D in primary care. Our results confirm this and suggest that Irish GPs are especially resistant to the development of a R&D culture. However, the R&D Culture Index can be a useful tool to identify groups in primary care that should be targeted in order to encourage research involvement. Once these groups have been targeted, any programme to encourage research involvement will need to take place in the context of protected time. The correlation of a high score in the R&D Culture Index with current research involvement in this study and with recent research activity in a previous study[6], suggests that the index functions as a marker of both research interest and activity[6]. A prospective study comparing scores from the R&D Culture Index with research activity would be useful to confirm its use in this respect. However, much variability in scoring remains unexplained and requires further investigation particularly in the case of GPs as a professional group. While not examined in this study, further to the generation of research within primary care, it is also essential to recognise the importance of the use of research in this sector of healthcare. Increased use of research within a practice may foster a culture of research, which may encourage research activity. Therefore, further studies regarding the use of research in practice would be of benefit.

## Conclusion

It may be unreasonable to assume that a culture supportive of research and development can be established rapidly; however, a culture that perceives clinical practice and research as entirely separate activities is unsustainable and outdated. This study helps to highlight the issues that need to be addressed in order to encourage such a cultural shift, primarily more research training and support particularly in basic research skills and to the opportunity to become research involved. Despite such challenges, primary healthcare professionals, given the appropriate environment, are willing and able to engage in R&D activity. This study also helps to identify a possible target population of professionals for any future R&D strategy, thus increasing the likelihood of any such R&D strategy achieving enduring success.

## Competing interests

The authors declare that they have no competing interests.

## Authors' contributions

LG (guarantor), COR, AMF, JN, AAI, DW, PC and AWM contributed to study conception and design. LG and COR were responsible for the acquisition of data while LG, COR, AMF, JN and AAI analysed the data and drafted the article. All authors revised the article and granted final approval to the version submitted for publication.

## Pre-publication history

The pre-publication history for this paper can be accessed here:


